# A Case of Uterine Hemorrhage

**Published:** 1850-05

**Authors:** Isaac E. Thayer

**Affiliations:** Grand Prairie, Wis.


### ARTICLE VI.

*A Case of Uterine Hemorrhage.*—By Isaac E. Thayer, M.D., of Grand Prairie, Wis.

On the evening of the 27th January, I was summoned to attend Mrs. E. H., in labor with her first child. On my arrival I found the uterine contractions irregular; os uteri but very little dilated, the soft parts in a rigid and unyielding state.—Accordingly I administered Tartarized Antimony 2 gr., Gum Opii 2 gr, and after the space of two hours the soft parts became quite yielding, and the labor advanced regularly until the head arrived at the inferior strait of the pelvis, where it met with some resistance on account of the large size of the head. I applied the forceps and delivered without much trouble. On examination I found the placenta adherent over its *whole* surface; the uterus was excited to contraction by external friction, and grappling the abdomen, which availed nothing in the expulsion of the placenta. I then proceeded to deliver, by introducing my hand and seperating its attachments, which resulted in a *profuse and alarming* hemorrhage, producing syncope in the space of three minutes. I immediately compressed the abdominal aorta firmly, while the contractions of the uterus were produced by the administration of fifteen grs. Ergot, and a continuous stream of cold water falling, the distance of eighteen inches upon the abdomen. The alarming symptoms soon subsided, and the mother and child are both doing well at this time.

